# The Role of Ecological and Complex Gait and Postural Tasks in the Assessment of Subtle Deficits in Parkinson’s Disease

**DOI:** 10.3390/healthcare14183108

**Published:** 2026-09-20

**Authors:** Patrizio Manzari, Amaranta Soledad Orejel Bustos, Andree Rossi, Giuseppe Vannozzi, Maria Gabriella Buzzi, Valeria Belluscio

**Affiliations:** 1 Department of Movement, Human and Health Sciences, University of Rome “Foro Italico”, P.zza Lauro de Bosis 15, 00135 Rome, Italy; patrizio.manzari00@gmail.com (P.M.); a.rossi11@studenti.uniroma4.it (A.R.); giuseppe.vannozzi@uniroma4.it (G.V.); 2 LIFE–Istituto Ricerca e Cura-Santa Lucia IRCCS, Via Ardeatina 306, 00179 Rome, Italy; a.orejel@hsantalucia.it (A.S.O.B.); mg.buzzi@hsantalucia.it (M.G.B.)

**Keywords:** inertial measurement units, neurological disease, balance, gait impairments, posturography, ecologically relevant assessment, gait harmony

## Abstract

**Background/Objectives**: Postural and gait assessment is essential in the clinical evaluation of people with Parkinson’s disease (PwPD). However, conventional clinical assessments may lack the complexity required to uncover subtle deficits, particularly in individuals who compensate well under controlled conditions but struggle in daily life. The aim of this study was to evaluate whether the incorporation of ecologically relevant postural and gait tasks into a controlled experimental environment enhances the detection of subtle motor deficits in PwPD, and to examine the ability of these tasks to distinguish between different levels of disease severity. **Methods**: Sixteen healthy subjects (HS) and 16 age-matched PwPD, further divided into Mild_PD *(n* = 9) and Moderate_PD (*n* = 7), performed a 60 s postural task on different surfaces (ground, medium elastic response, pebbles and root-like) and two walking tasks on ground (10-meter walk test, 10MWT) and on a 4 m mat with medium elastic response (MatWalk), while wearing five inertial measurement units. Postural and spatiotemporal parameters were extracted and statistically analyzed. **Results**: Postural task analysis revealed limited between-group differences, whereas within-group comparisons identified several significant differences across surfaces in HS and fewer in the PD group. Gait task analysis showed broader impairments, with some parameters differing in both walking tests and others only during the MatWalk when comparing HS and PD. Compliant surface walking was particularly sensitive to subtle gait alterations in Mild_PD. **Conclusions**: Ecologically relevant walking tasks, especially on compliant surfaces, may complement conventional assessments by revealing subtle gait impairments in PwPD not fully captured by standard clinical tests.

## 1. Introduction

Parkinson’s disease (PD) is characterized by dopamine deficiency, leading to a broad spectrum of motor and non-motor symptoms, including impaired sleep quality, significant cognitive deterioration, and gait and balance disorders [1,2]. Reduced basal ganglia output to the supplementary motor area and premotor cortex contributes to a mismatch between the intended and executed amplitudes of well-learned, repetitive motor sequences [3]. Balance control relies on the brain’s continuous integration of internal and external sensory information during both movement (dynamic balance) and standing still (static balance) [4].

In people with PD (PwPD), reduced peripheral sensation arising from degeneration of cutaneous receptors and peripheral sensory nerves may exacerbate balance impairments [5]. Uneven or irregular surfaces can further reduce the reliability of somatosensory feedback from the feet and lower limbs, challenging the ability to adapt their gait, and leading to changes in body kinematics, spatiotemporal gait parameters, and postural control, other than increasing the risk of falls. Notably, even among healthy young adults, walking on uneven terrain has been shown to alter lower-limb and trunk kinematics, as well as gait characteristics and their variability [6]. Given that daily mobility often requires individuals to stand or walk on different surfaces (e.g., grass, cobblestone), the ability to effectively adapt to changing environmental demands is essential for safe and efficient mobility. However, PwPD exhibits a reduced capacity to adjust to progressively challenging balance tasks or conditions compared with healthy individuals, likely because of disease-related bradykinesia and rigidity [7].

Despite substantial advances in the characterization of postural and gait impairments in PwPD, clinical assessment remains largely reliant on standardized rating scales, such as the Movement Disorder Society–Unified Parkinson’s Disease Rating Scale (MDS-UPDRS) and the Dynamic Gait Index (DGI). Quantitative evaluations are often complemented with Inertial Measurement Units (IMUs), wearable sensors capable of providing objective measures of gait and balance during standardized clinical tests. Although these approaches are validated and widely adopted, they are typically implemented through standardized motor assessments that may be insufficiently challenging to reveal subtle impairments in gait and postural control.

Previous biomechanical studies have shown that PwPD adapts their gait when walking on different surfaces. Compared with regular or firm surfaces, irregular and compliant surfaces have been associated with greater reductions in gait speed, cadence, and step length, as well as changes in lower-limb kinematics and whole-body dynamic stability [8,9]. These findings suggest that surface characteristics represent an important environmental constraint for gait in PwPD, requiring adaptations in both spatiotemporal and biomechanical gait parameters. However, to the best of the author’s knowledge, only a few studies have systematically investigated gait adaptations to different surface conditions, while the effects of different surfaces on postural control have received comparatively less attention. Consequently, the effects of surface characteristics on gait and postural control remain insufficiently understood.

This limited evidence underscores the need for more comprehensive and ecologically relevant assessment paradigms that incorporate functional tasks and environmental challenges representative of everyday life, as the different surfaces that PwPD can encounter in daily life. The inclusion of such assessments may be essential, given that neglecting surface-related environmental demands could lead to the omission of clinically relevant factors associated with postural instability and fall risk [10]. Furthermore, conventional walking and postural clinical assessments may lack sufficient sensitivity to detect subtle differences in motor performance across disease stages, potentially limiting their ability to discriminate between different levels of disease severity, particularly in individuals with milder disease.

Accordingly, the present study was designed to evaluate whether the incorporation of ecologically relevant motor and postural tasks within a controlled experimental environment could improve the identification of functional impairments and subtle motor deficits in PwPD. A secondary objective was to examine the ability of these tasks to distinguish between different levels of disease severity.

We hypothesized that the introduction of ecologically relevant and challenging surface conditions would elicit greater gait and postural adaptations in PwPD than conventional surfaces, allowing for the detection of subtle motor impairments that may not be captured under standard conditions. We further hypothesized that these adaptations would become more pronounced with increasing disease severity, with participants with moderate PD showing greater impairment than those with mild PD.

## 2. Materials and Methods

The present study was approved by the ethical committee of LIFE–Istituto Ricerca e Cura e Santa Lucia IRCCS in Rome, Italy (approval number: CE/2022_040). All participants provided their written informed consent for participating in the study.

### 2.1. Participants

Sixteen healthy subjects (HS) (age: 64.63 ± 8 years; 12 females and 4 males) and 16 PwPD (age: 70 ± 7 years; 4 females and 12 males) were involved in the study. This sample size was set based on an a priori power analysis performed using G*Power (version 3.1.9.7): assuming a medium effect size (f = 0.25), a significance level of α = 0.05, and a statistical power of 0.80, a total sample size of 32 participants (16 per group) was required for the comparison between HS and PwPD. The calculation was based on a repeated measures within–between interaction design, in which group was considered the between-subject factor and surface condition the within-subject factor, using 4 as number of conditions. The inclusion criteria for HS were (i) age range 18–85 years, (ii) absence of neurological and motor deficits, (iii) absence of injuries which could influence motor performance in the previous 6 months. Inclusion criteria for PwPD were (i) age range: 40–85 years; (ii) Mini-mental State Examination (MMSE) score > 24; (iii) Hoehn & Yahr (H&Y) score: 1–3. Exclusion criteria for all participants were (i) severe unilateral spatial neglect (diagnosed with Letter Cancellation test, the Barrage test, the Sentence Reading test, and the Wundt–Jastrow Area Illusion Test), (ii) severe aphasia (diagnosed with neuropsychological assessment), (iii) alcohol or substance abuse, (iv) MMSE score < 24. PwPD underwent the evaluation in the ON medication state (the best subjective motor state between 30–45 min from the assumption of the usual dose of levodopa).

Furthermore, to codify for different levels of motor symptoms, PwPD were further stratified into lower and higher motor impairment groups based on MDS-UPDRS-Part III scores: participants with a score ≤ 27 were considered less severe (9 people, Mild_PD, age 67.89 ± 8 years), while those with a score > 27 were considered more severe (7 people, Moderate_PD, age 72.71 ± 5 years), according to Martínez-Martín et al. [11]. Also in this case, an a priori sample size calculation was performed assuming three groups (HS, Mild_PD, and Moderate_PD), and four repeated measurements corresponding to the four surface conditions of the postural task. Assuming a medium effect size (f = 0.25), a significance level of α = 0.05, a statistical power of 0.80, a correlation of 0.50 among repeated measures, and a nonsphericity correction of 1, the required total sample size was estimated to be 30 participants. The final sample included therefore 32 participants. Although the three groups were not equally sized, the total sample size was above the a priori requirement.

### 2.2. Procedures

#### 2.2.1. Clinical Assessment

The following clinical scales were administered by an expert physiotherapist to all participants to assess static and dynamic balance, falls and ambulation skills:

- Dynamic Gait Index (DGI), used to assess the subject’s ability to modify his/her gait in response to changing task demands. It consists of items rated from 0 to 3 (0 = severely impaired; 3 = normal performance), yielding a maximum score of 24 points [12,13].
- Mini-Balance Evaluation Systems Test (Mini-BEST), which assesses vestibular and non-vestibular balance, gait, and functional mobility, yielding a maximum score of 28 points. It consists of items rated from 0 (the lowest level of function) to 2 (indicates the highest level of function), yielding a maximum score of 28 points [14,15].

In addition, PwPD were also assessed using the MDS-UPDRS-Part III, to explore motor functionality through 18 items [16], and the H&Y scale, a five-stage clinical staging system recommended for describing disease severity and classifying patients according to the progression of PD [17].

A Postural Instability/Gait Dominant (PIGD) related subscore (partial axial/gait subscore) was then calculated from the MDS-UPDRS-Part III by summing items 3.10 (gait), 3.11 (freezing of gait), and 3.12 (postural stability), resulting in a score ranging from 0 to 12 [18].

Records of fall history were obtained for all participants to collect detailed event-based information related to fall events in the 12 months before the assessment. In detail, past year fall status, total number of falls, fall severity of injury (whether any fall resulted in medical care or physical trauma), primary contributing factor (e.g., balance loss, trip hazard, room-specific hazard/environmental, slipped, while turning, etc.), and location (e.g., indoor, outdoor) were the information collected.

#### 2.2.2. Motor Assessment

Each participant was asked to perform four static posturography and two walking tasks on different surfaces, all performed in a randomized order. Before the test began, participants received a careful explanation and demonstration of the tasks they were expected to perform, along with explicit directions from the instructor on when to start and finish.

**Figure 1:**
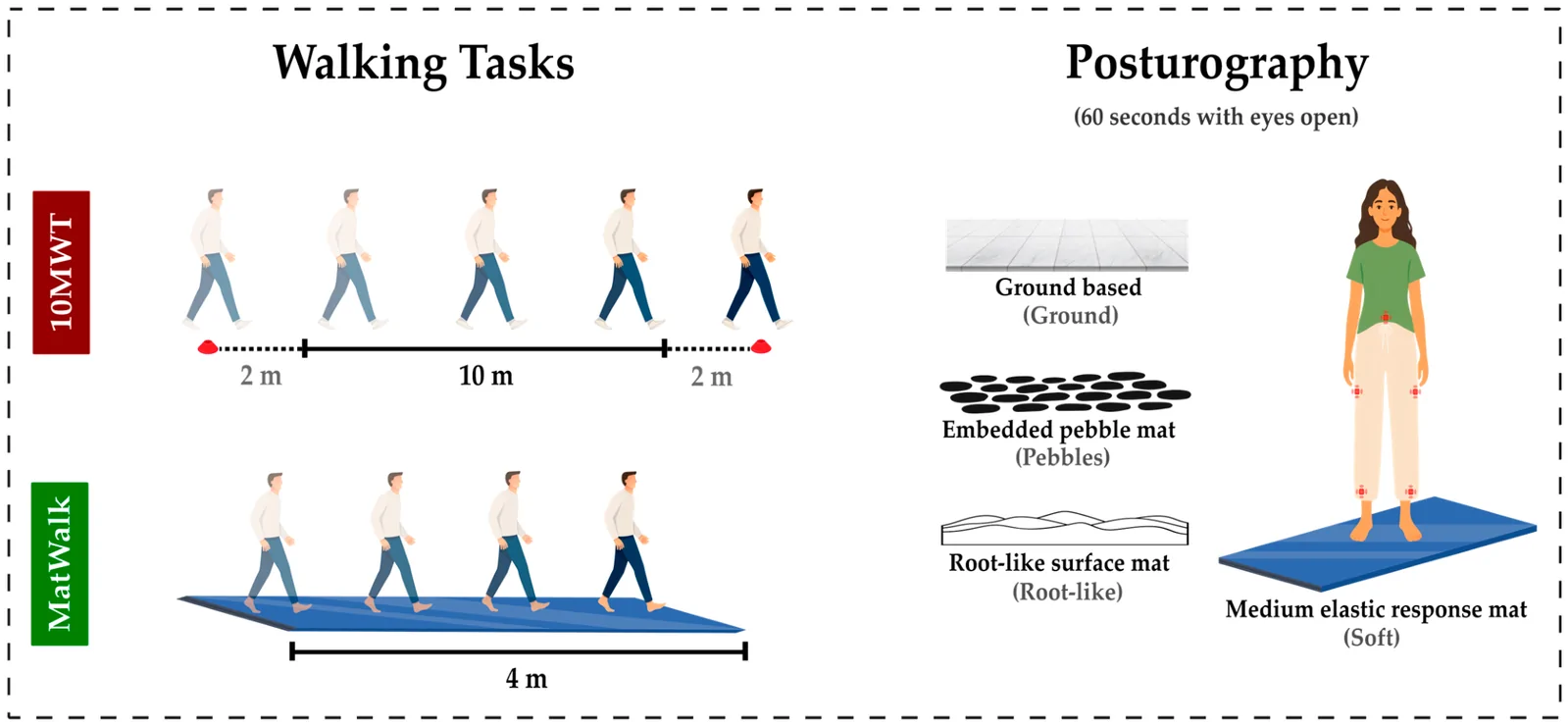
Graphical representation of the walking and posturography tasks included in the assessment protocol. 10MWT = 10-Meter Walk Test.

The static posturography tasks were conducted on the ground and on three different mats (width 200, depth 54, height 7 cm, Human Tecar^®^, Synergy Mat, Lecco, Italy) [19]. Specific features for each mat are reported in Table 1. Each task lasted 60 s, with the participant standing barefoot, feet shoulder-width apart, focusing on a visual reference mark placed in front of them at a two-meter distance with eyes open. The four surfaces were the ground (Ground), a medium elastic response mat (Soft), an embedded pebble mat (Pebbles), and a root-like surface mat (Root-Like) (Figure 1).

The two walking tasks were a 10-Meter Walk Test (10MWT), consisting of a 14 m self-paced walking, repeated three times, in a hallway wearing comfortable shoes, where the first and the last two meters were removed from the analysis to consider only steady-state walking, and a MatWalk, which consists of walking barefoot back and forth on a 4 m long soft mat with only the straight-walking portions considered for the analysis, while turning and acceleration/deceleration phases were excluded. For the MatWalk, central 2 strides were therefore retained for the analysis. Participants walk at their own pace, performing five repetitions. Therefore, each participant performed three trials of straight walking over a 10 m overground path and five trials of straight walking over a 4 m mat. For each participant and for each walking condition, the values obtained across the repeated trials were checked for outliers, averaged, resulting in a single representative value for each gait parameter and condition. Thus, each participant contributed one value per parameter for each walking condition to the subsequent statistical analyses.

Throughout the entire duration of the assessment, (approximately 60 min, including the administration of the clinical scales), an expert physiotherapist stood near participants to prevent falls. Rest periods were provided between tasks to minimize fatigue, and the duration of each rest period was about 2 min or longer if needed. 

### 2.3. Equipment

**Figure 2:**
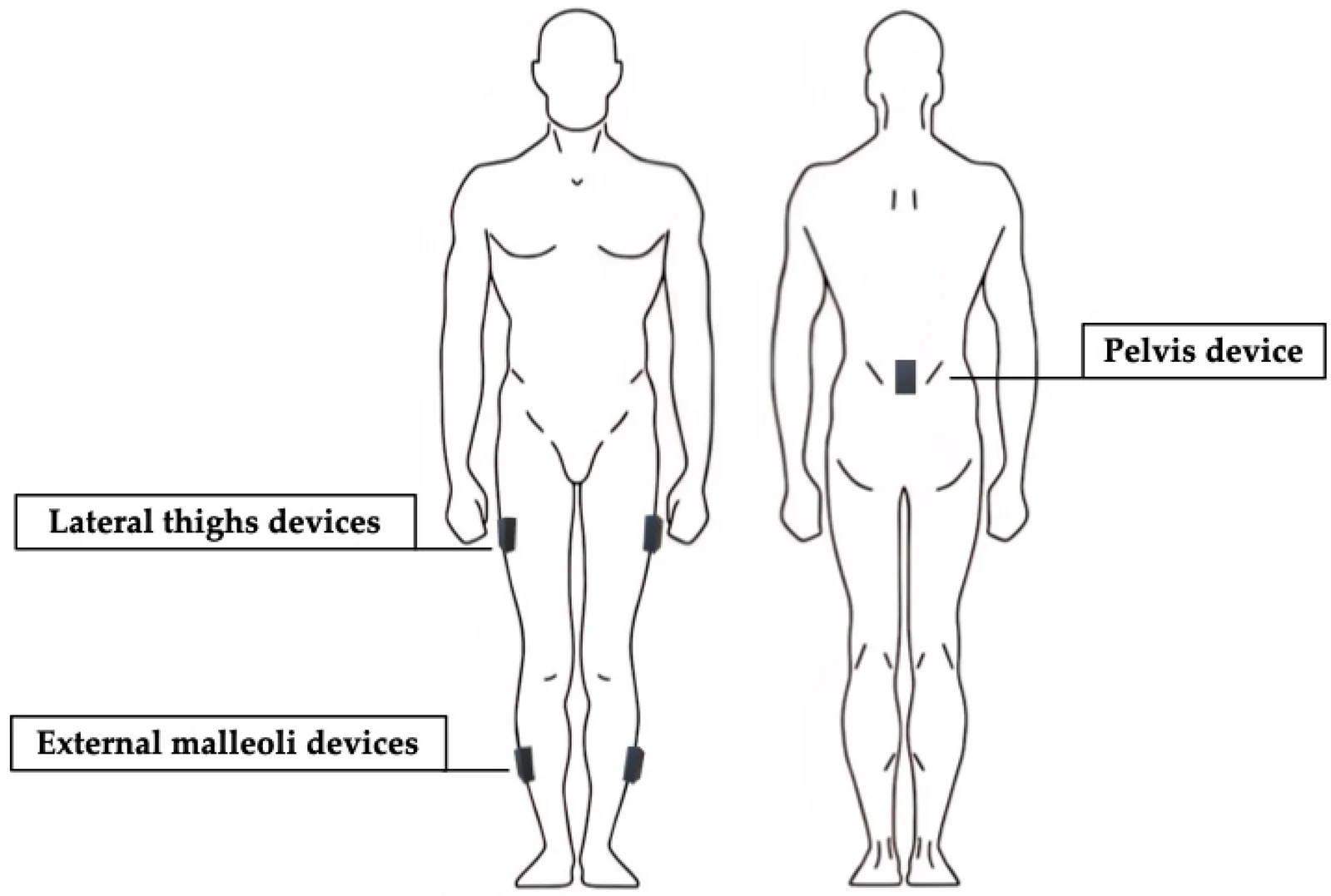
IMUs set-up.

During the assessment, participants wore five automatically synchronized IMUs (100 Hz, Captiks srl, Guidonia Montecelio, Italy) positioned slightly above the pelvis (L4–L5 level), and on both lateral thighs and above the external malleoli (Figure 2). Each IMU was securely fixed to the participant’s body with Velcro straps.

### 2.4. Data Processing

Data processing was performed using Movit Analyzer algorithms [20]: the software-derived parameters have been previously validated against an optoelectronic motion-capture system [20]. Specific mathematical details of the proprietary algorithms are not publicly available, but gait events were identified using heel strike and toe off.

The following parameters were considered as predefined primary outcomes: the Root Mean Square (RMS, in mm) for postural tasks and the stance/swing ratio for walking tasks. 

The RMS was used to quantify postural oscillations, the Center of Mass variability around the mean Center of Mass (CoM) trajectory. It represents the displacement of the pelvic sensor around the mean of the pelvic sensor trajectory. Right–Left Stance/Swing Ratio is the ratio of the duration of the stance phase to that of the swing phase of the same foot, providing complementary information on the spatiotemporal organization of gait [21]. It has been shown to approximate the Golden Ratio (φ ≈ 1.618), an irrational number widely observed in physical and biological systems. This characteristic has been referred to as “gait harmony” and has been proposed as a marker of physiological gait organization, highlighting the smooth “melodic” flow of movement [22,23].

From the pelvis IMU, two additional parameters were computed for postural tasks:

- Ellipse Area (mm^2^), the distance covered by the pelvic sensor following postural oscillations.
- Mean Speed (mm/s), the mean value of the speed related to the displacement of the pelvic sensor.

The following secondary parameters were considered for the two walking tasks:

- Cadence (step/min), number of steps taken in one minute, taking into account the steps taken by both feet.
- Gait Velocity (m/s), average walking speed maintained during the trial.
- Step Length (m), the distance covered in a single step, measured from the heel strike of one foot to the heel strike of the opposite foot.

### 2.5. Statistical Analysis

Descriptive statistical analyses were performed using R Studio (v 4.6.0, Posit Software, PBC, Vienna, Austria), and the alpha level of significance was set at 0.05. The normal distribution of each parameter was verified using the Shapiro–Wilk test. Post hoc analyses were performed using the Bonferroni correction method to prevent Type 1 Error. The following parametric and non-parametric tests were performed:

- *t*-test or Mann–Whitney U test to investigate if significant differences existed between HS and PwPD. The assumption of equal variances was tested using Levene’s Test.
- One-way ANOVA or Kruskal–Wallis test to investigate if significant differences existed between the HS, Mild_PD and Moderate_PD. Post hoc analysis for the Kruskal–Wallis was performed with a Mann–Whitney U test.
- Repeated measures ANOVA or Friedman and Wilcoxon Test to determine whether there were significant differences in postural and walking parameters among the four different surfaces.

The multiplicity correction was applied within each predefined family of pairwise comparisons associated with a significant omnibus test, rather than across all dependent variables and experimental conditions. This approach was selected to control the family-wise error rate within each specific set of post hoc comparisons while preserving the exploratory analysis of the different gait and postural parameters.

No data imputation was performed. Analyses were performed using the available observations for each parameter, so the effective sample size could vary across within-group and between-group analyses. The sample-size estimation was originally based on a within–between interaction framework, reflecting the expected effect of group and task condition. In the present exploratory analysis, however, the available data were analyzed using separate within-group and between-group comparisons. Observed statistically significant differences in the response pattern between/among groups were therefore considered as exploratory.

## 3. Results

### 3.1. Demographic Characteristics and Clinical Scales Score Results

Demographic and anthropometric characteristics and clinical scale scores for HS, PwPD and the two clinically different PD subgroups are reported in Table 2. Results showed no statistically significant differences for the factors “age”, “body mass” and “height” among the groups. Moderate_PD group (defined as the more severe; see Methods) presented statistically significant worse scores in the MDS-UPDRS-Part III compared to Mild_PD, as expected since used for the motor functionality classification. In comparison with HS, who completed only DGI and Mini-BEST scales, no statistically significant differences on the clinical scales were found for the Mild_PD group, whereas the Moderate_PD group showed significant differences for both DGI and Mini-BEST scales. In the Mild group, seven patients had left-sided involvement and one patient had bilateral involvement, whereas all patients in the Moderate group had bilateral involvement.

Descriptive statistics for fall history data from 32 participants across the three groups HS (*n* = 16), Mild_PD (*n* = 9) and Moderate_PD (*n* = 7) are summarized in Table 3. Of the 32 participants with available fall status data, seven (21.9%) reported at least one fall in the past year. The proportion of participants reporting falls was 12.5% in healthy controls (2/16), 22.2% in the Mild_PD group (2/9), and 42.9% in the Moderate_PD group (3/7).

Furthermore, missing data were limited and unevenly distributed across groups and parameters; they occurred when the analysis software was unable to derive a valid estimate for the corresponding parameter from the acquired signal. The number of missing observations for each affected variable and study group is reported in Table 4.

### 3.2. Motor Assessment Results 

#### 3.2.1. Posturography Tasks

**Figure 3:**
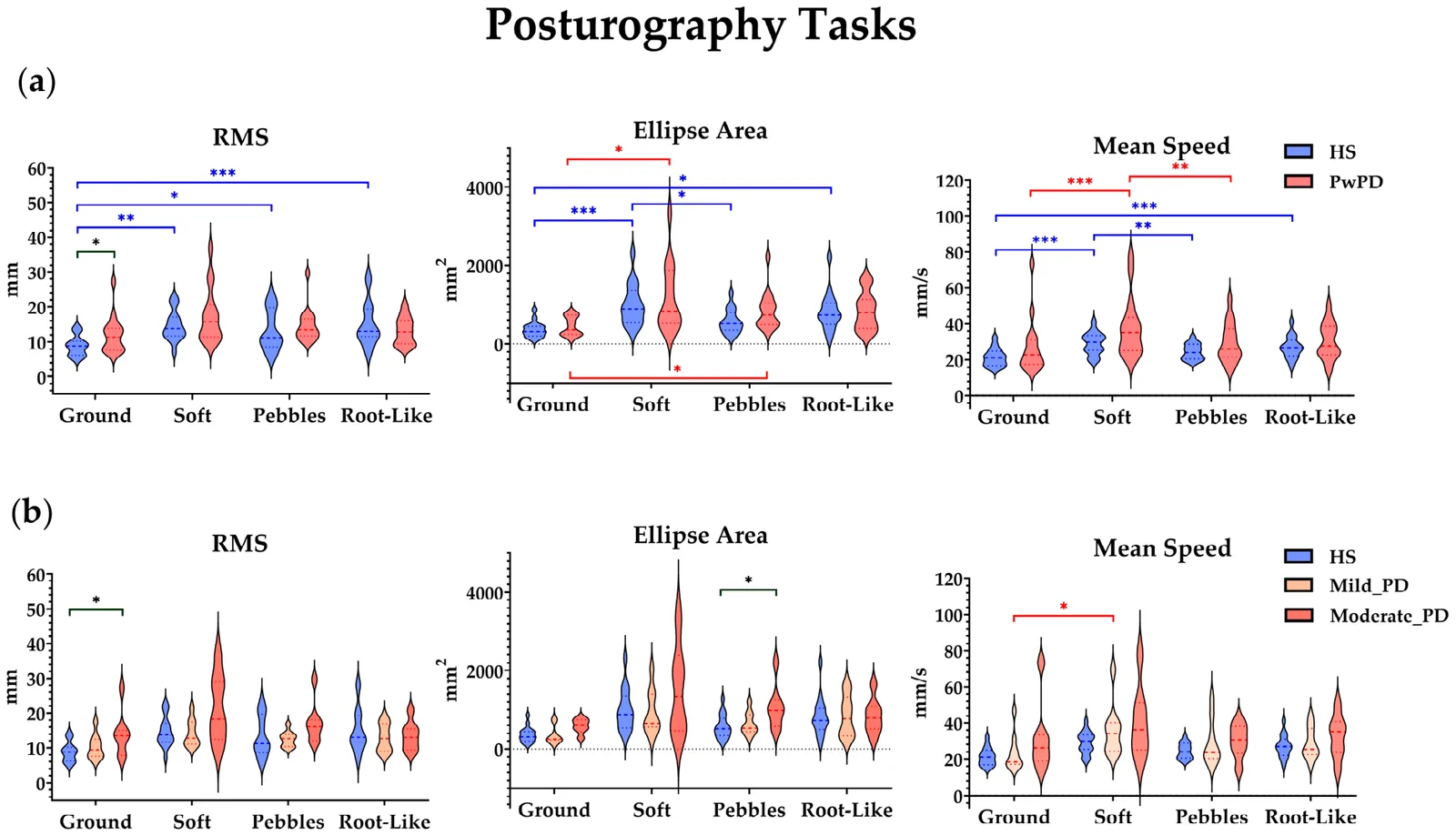
Violin plots of the postural parameters. The upper section shows the comparisons between HS and PwPD (**a**), and the lower section displays comparisons among HS, Mild_PD and Moderate_PD (**b**). Horizontal lines and asterisks indicate statistically significant differences (* for *p* < 0.05, ** for *p* < 0.01, *** for *p* < 0.001). Black-colored lines indicate differences between HS and PwPD; whereas blue-colored lines indicate differences within-group comparisons for HS, and red-colored lines for PwPD. Within each violin plot, the dashed central line denotes the median of the data, while the two outer dotted lines correspond to the first (25th) and third (75th) quartiles, respectively.

Violin plots of extracted parameters from the posturography task on different surfaces are shown in Figure 3. The upper section reports the comparisons between HS and PwPD (Figure 3a), while the lower section shows the comparisons among HS, Mild_PD and Moderate_PD (Figure 3b). The RMS parameter revealed a significant difference between HS (*Mdn* = 8.87, *IQR* = 6.41–9.93) and PwPD (*Mdn* = 12.36, *IQR* = 8.04–14.18) during the postural task performed on the Ground (*U* = 70, *Z* = −2.186, *p* = 0.029, *r* = 0.38). No further statistically significant differences were observed between HS and PwPD on the other surface conditions. Analysis of within-group comparisons showed that HS presented higher sensitivity to changes in surface characteristics. More specifically, a one-way repeated measures ANOVA determined that the RMS differed significantly in HS across the testing surfaces. Mauchly’s test indicated that the assumption of sphericity had been violated χ^2^(5) = 11.155, *p* = 0.049; therefore, a Greenhouse–Geisser correction was used, *F*(1.927, 28.912) = 7.081, *p* = 0.003, $\eta_{p}^{2}$ = 0.32. Post hoc pairwise comparisons with Bonferroni correction showed that RMS significantly increased from Ground to Soft surface (*M* = 8.85, *SD* = 2.89; and *M* = 14.64, *SD* = 4.30, respectively; *p* = 0.002), from Ground to Pebbles (*M* = 13.29, *SD* = 5.85, *p* = 0.031), and from Ground to Root-like condition (*M* = 15.08, *SD* = 6.41, *p* < 0.001). Similarly, the Ellipse Area also showed significant differences across the testing surfaces. Mauchly’s test indicated that the assumption of sphericity had been violated *χ*^2^(5) = 23.150, *p* < 0.001; therefore, a Greenhouse–Geisser correction was used, *F*(1.635, 24.531) = 7.952, *p* = 0.004, $\eta_{p}^{2}$ = 0.35. Post hoc tests with Bonferroni correction showed that Ellipse Area significantly increased from Ground to Soft surface (*M* = 360.63, *SD* = 197.47; and *M* = 991.16, *SD* = 530.52, respectively; *p* < 0.001), from Ground to Root-like (*M* = 779.18, *SD* = 523.53, *p* = 0.022), and decrease from Soft to Pebbles (*M* = 991.16, *SD* = 530.52; and *M* = 562.83, *SD* = 305.91, respectively; *p* = 0.018). Significant differences were also found for the Mean Speed parameter. Mauchly’s test indicated that the assumption of sphericity was satisfied, (*χ*^2^(5) = 3.732, *p* = 0.589), *F*(3, 45) = 15.413, *p* < 0.001, $\eta_{p}^{2}$ = 0.51. Post hoc tests with Bonferroni correction showed that Mean Speed scores significantly increased from Ground to Soft surface (*M* = 21.33, *SD* = 4.94; and *M* = 29.01, *SD* = 6.12, respectively; *p* < 0.001), from Ground to Root-like (*M* = 26.94, *SD* = 6.18, *p* < 0.001), and decreased from Soft to Pebbles condition (*M* = 29.01, *SD* = 6.12; and *M* = 24.46, *SD* = 4.32, respectively; *p* = 0.002 (Figure 3a).

In contrast, there were no significant differences across surface conditions within the PwPD group for the RMS, while significant differences were found in the Ellipse Area (*χ*^2^(3) = 9.480, *p* < 0.001, *W* = 0.21). Post hoc analysis with Wilcoxon signed-rank tests with Bonferroni correction showed that Ellipse Area scores significantly differed between Ground and Soft surfaces (*Mdn* = 354.63, *IQR* = 242.24–694.42; and *Mdn* = 823.45, *IQR* = 556.03–1660.72, respectively; *p* = 0.040), and between Ground and Pebbles (*Mdn* = 728.94, *IQR* = 507.12–997.50, *p* = 0.026). Other significant differences were also found in the Mean Speed parameter (*χ*^2^(3) = 23.025, *p* < 0.001, *W* = 0.48). Post hoc analysis with Wilcoxon signed-rank tests with Bonferroni correction showed differences between Ground and Soft conditions (*Mdn* = 22.80, *IQR* = 17.61–31.05; *Mdn* = 35.19, *IQR* = 25.48–41.33; *p* < 0.001), and between Soft and Pebbles (*Mdn* = 35.19, *IQR* = 25.48– 41.33; *Mdn* = 26.17, *IQR* = 22.70–35.57; *p* = 0.008). For the comparison among groups (*HS, Mild_PD and Moderate_PD)*, statistically significant differences were observed for the RMS parameter (*χ*^2^(2) = 8.339, *p* = 0.015, *η*^2^ = 0.18) between HS (*Mdn* = 8.87, *IQR* = 6.41–9.93) and Moderate_PD (*Mdn* = 13.94, *IQR* = 13.28–14.96) during the Ground condition (*p* = 0.020). Likewise, the Ellipse Area showed a significant difference (*F*(2, 29) = 4.549, *p* = 0.035, *η*^2^ = 0.24) when comparing HS with Moderate_PD (*M* = 562.83, *SD* = 305.91; *M* = 1066.18, *SD* = 573.19; *p* = 0.018) on the Pebbles surface (Figure 3b). Finally, there was a significant difference across surfaces within the Mild_PD group for the Mean Speed parameter (*χ*^2^(3) = 8.600, *p* < 0.035, *W* = 0.32). Post hoc analysis with Wilcoxon signed-rank tests with Bonferroni correction showed differences between Ground and Soft conditions (*Mdn* = 18.31, *IQR* = 17.61–24.03; *Mdn* = 34.17, *IQR* = 25.60–40.15; *p* = 0.023).

#### 3.2.2. Walking Tasks

**Figure 4:**
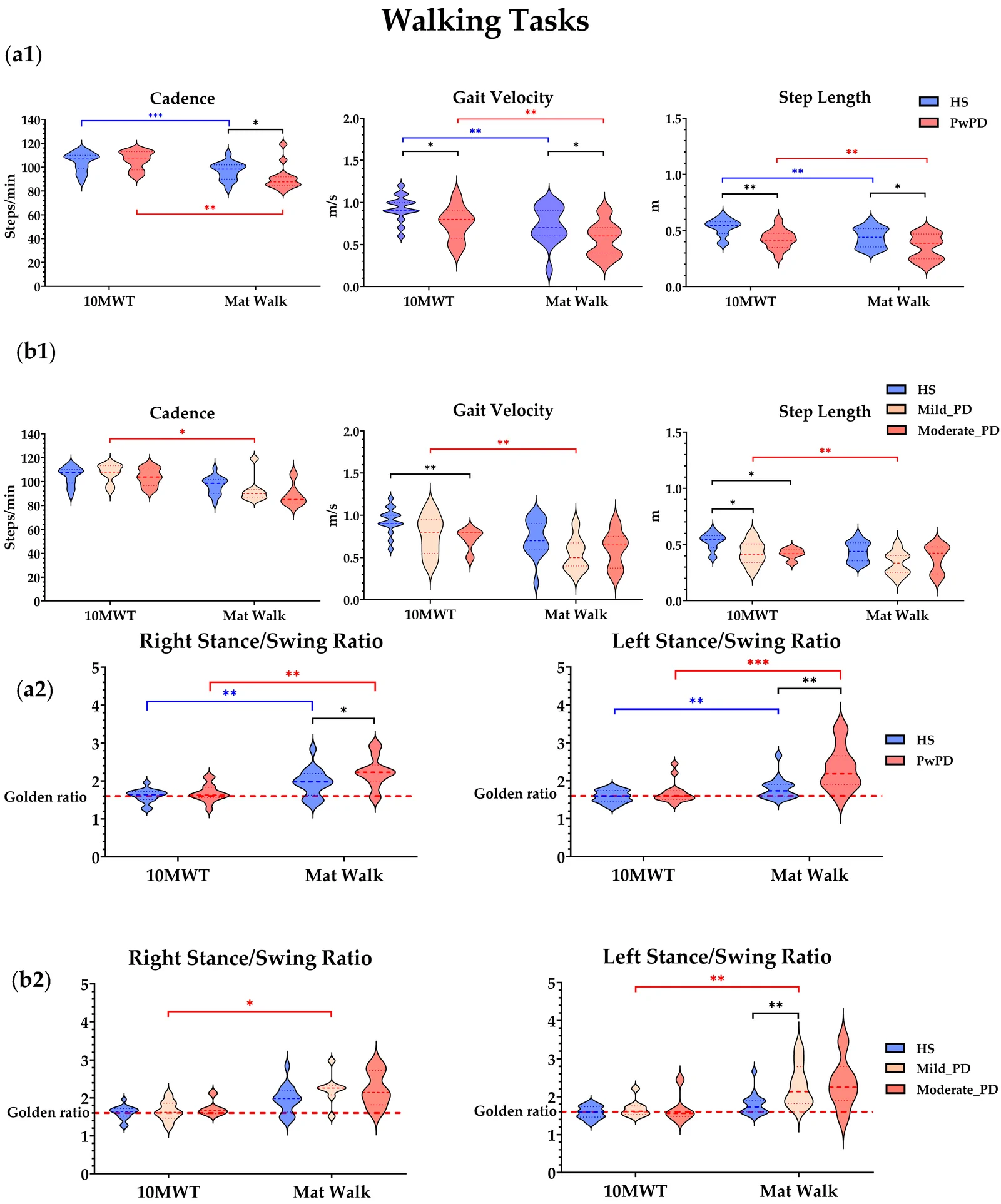
Violin graph of the Walking parameters. The upper section shows the comparisons between HS and PwPD (**a1**,**a2**), and the lower section displays comparisons among HS, Mild_PD and Moderate_PD (**b1**,**b2**). Horizontal lines and asterisks indicate statistically significant differences (* for *p* < 0.05, ** for *p* < 0.01, *** for *p* < 0.001). Black-colored lines indicate differences between HS and PwPD, whereas blue-colored lines indicate differences within-group comparisons for HS, and red-colored lines for PwPD. The Stance Swing graphs feature a horizontal-red-dotted line, which helps identify the golden ratio, or the optimal value for the ratio between stance and swing. Within each violin plot, the dashed central line denotes the median of the data, while the two outer dotted lines correspond to the first (25th) and third (75th) quartiles, respectively.

Violin plots of parameters extracted from walking tasks are shown in Figure 4. The upper section reports the comparisons between HS and PwPD (Figure 4a), while the lower section shows comparisons among HS, Mild_PD, and Moderate_PD (Figure 4b). Significant differences were found between HS and PwPD. Notable variations were observed in the Cadence parameter during the MatWalk test. The HS group presented significantly higher number of steps per minute (*Mdn* = 98.45, *IQR* = 90.75–101.63) than the PwPD group (*Mdn* = 87.75, *IQR* = 84.83–91.18), *U* = 57, Z = 2.286, *p* = 0.022, *r* = 0.47. Additionally, there were statistically significant variations in Gait Velocity between the two walking tasks. More specifically, in the 10MWT, the HS group showed higher walking speed (*M* = 0.93, *SD* = 0.15) than the PwPD group (*M* = 0.75, *SD* = 0.20), equal variances were assumed (*F* = 1.118, *p* = 0.300), *t*(27) = 2.783, *p* = 0.010, *d* = 1.03. The same pattern was observed in the MatWalk task, with HS who displayed a higher walking speed (*M* = 0.73, *SD* = 0.22) than PwPD (*M* = 0.56, *SD* = 0.20), equal variances were assumed (*F* = 0.006, *p* = 0.938), *t*(28) = 2.162, *p* = 0.039, *d* = 0.79. Similar results were found also in the Step Length in both the 10MWT (HS: *M* = 0.53, *SD* = 0.07, PwPD: *M* = 0.42, *SD* = 0.09), equal variances were assumed (*F* = 0.308, *p* = 0.583), *t*(27) = 3.623, *p* = 0.001, *d* = 1.35, and in the MatWalk task (HS: *M* = 0.44, *SD* = 0.09; PwPD: *M* = 0.35, *SD* = 0.11), equal variances were assumed (*F* = 2.281, *p* = 0.142), *t*(28) = 2.319, *p* = 0.028, *d* = 0.85. Finally, there were statistically significant differences in the Right and Left Stance/Swing Ratio during the MatWalk test. The HS group presented significantly lower both Right and Left ratios compared to PwPD (HS_Right: *Mdn* = 1.98, *IQR* = 1.74–2.11; PwPD_Right: *Mdn* = 2.23, *IQR* = 2.06–2.32), *U* = 61, *Z* = −2.120, *p* = 0.033, *r* = 0.40; HS_Left: *Mdn* = 1.73, *IQR* = 1.60–1.89; PwPD_Left: *Mdn* = 2.19, *IQR* = 1.95–2.55), *U* = 42, *Z* = −2.910, *p* = 0.006, *r* = 0.53 (Figure 4(a2)).

Within the HS group, statistically significant differences were also found between the walking tasks. The Cadence parameter showed significant defenses between the 10MWT (*M* = 105.15, *SD* = 7.07) and the MatWalk (*M* = 96.72, *SD* = 7.55), *t*(15) = 5.841, *p* < 0.001, *d* = 1.15, as well as the Gait Velocity (10MWT: *M* = 0.93, *SD* = 0.15; MatWalk: *M* = 0.73, *SD* = 0.22), *t*(14) = 3.477, *p* = 0.004, *d* = 0.90. Likewise, differences were also found for the Step Length between the 10MWT (*M* = 0.53, *SD* = 0.07) and the MatWalk (*M* = 0.44, *SD* = 0.09), *t*(14) = 3.925, *p* = 0.002, *d* = 1.01. And lastly, the Right and Left Stance/Swing Ratio differed between the 10MWT (*Mdn* = 1.64, *IQR* = 1.53–1.72 and *Mdn* = 1.60, *IQR* = 1.47–1.73, respectively) and the MatWalk (*Mdn* = 1.98, *IQR* = 1.74–2.11 and *Mdn* = 1.73, *IQR* = 1.60–1.89, respectively), *Z* = −2.637, *p* = 0.006, *r* = 0.75.

Similar findings were observed within the PD group in all walking parameters. More specifically, statistically significant differences were found in Cadence between the 10MWT (*Mdn* = 107.60, *IQR* = 100.40–112.95) and the MatWalk (*Mdn* = 87.75, *IQR* = 84.83–91.18), *Z* = 2.900, *p* = 0.004, *r* = 0.67, Gait Velocity (10MWT: *M* = 0.75, *SD* = 0.20; MatWalk (*M* = 0.56, *SD* = 0.20), *t*(11) = 3.728, *p* = 0.003, *d* = 0.93, Step Length (10MWT: *M* = 0.42, *SD* = 0.09; MatWalk task: *M* = 0.35, *SD* = 0.11), *t*(11) = 3.566, *p* = 0.004, *d* = 0.69, and for both the Right and Left Stance/Swing Ratios between the 10MWT (*Mdn* = 1.63, *IQR* = 1.57–1.78 and *Mdn* = 1.60, *IQR* = 1.52–1.71, respectively) and the MatWalk (*Mdn* = 2.23, *IQR* = 2.06–2.32; and *Mdn* = 2.19, *IQR* = 1.95–2.55, respectively), with *Z* = −2.760, *r* = 0.87, *p* = 0.003 for the Right, and *Z* = −3.180, *r* = 1, *p* < 0.001 for the Left.

When considering the analysis of comparisons among HS and the two PD subgroups, the analyses revealed statistically significant differences for the Step Length on the 10MWT, *F*(2, 26) = 6.342, *p* = 0.006, *η*^2^ = 0.33. Bonferroni post hoc comparisons revealed that HS (*M* = 0.53, *SD* = 0.07) reported significantly higher Step Length than both the Mild_PD group (*M* = 0.43, *SD* = 0.11), *p* = 0.016, and the Moderate_PD group (*M* = 0.42, *SD* = 0.05), *p* = 0.038. The Mild_PD group and Moderate_PD group did not significantly differ from each other, *p* > 0.05. Also, statistically significant differences were found between groups and Gait Velocity in the 10MWT, *χ*^2^(2) = 7.097, *p* = 0.029, *η*^2^ = 0.20. Post hoc analysis with Mann–Whitney tests with Bonferroni correction showed that only HS (*Mdn* = 0.90, *IQR* = 0.90–1.00) reported significantly higher walking speed than that of the Moderate_PD (*Mdn* = 0.80, *IQR* = 0.70–0.80), *p* = 0.04. On the other hand, during the MatWalk task, only significant differences were found for the Left Stance/Swing Ratio between the groups, *χ*^2^(2) = 8.470, *p* = 0.014, *η*^2^ = 0.24. Post hoc analysis with Bonferroni correction revealed that only HS (*Mdn* = 1.73, *IQR* = 1.60–1.89) reported significantly lower stance-to-swing phase ratio for left foot than that of the Mild_PD group (*Mdn* = 2.13, *IQR* = 1.90–2.57), *p* = 0.007.

Within-group analyses in the Mild_PD group, statistically significant differences were found for all the walking parameters comparison between 10MWT and MatWalk. Specifically, the Cadence parameter (*Mdn* = 107.90, *IQR* = 106.30–113.20 and *Mdn* = 89.85, *IQR* = 86.60–91.90, respectively), *Z* = 2.240, *p* = 0.03, *r* = 0.79; Gait Velocity (*M* = 0.77, *SD* = 0.23 and *M* = 0.54, *SD* = 0.20, respectively), *t*(7) = 4.461, *p* = 0.003, *d* = 1.05; Step Length (*M* = 0.43, *SD* = 0.11 and *M* = 0.34, *SD* = 0.10, respectively), *t*(7) = 4.324, *p* = 0.003, *d* = 0.87 and the Right (*Mdn* = 1.62, *IQR* = 1.52–1.84; *Mdn* = 2.26, *IQR* = 2.16–2.29, respectively), *Z* = −2.100, *p* = 0.039, *r* = 0.74; and Left Stance/Swing Ratios (*Mdn* = 1.61, *IQR* = 1.55–1.74 and *Mdn* = 2.13, *IQR* = 1.90–2.57, respectively), *Z* = −2.521, *p* = 0.008, *r* = 0.89. Lastly, no significant within-group differences were found in the Moderate_PD group.

## 4. Discussion

The aims of the study were to determine whether the integration of gait and postural tasks inspired by real-life contexts could improve the identification of functional impairments and subtle deficits in PwPD, and to establish whether such tasks could be used to distinguish between different levels of disease severity.

With respect to the initial hypotheses of the study, findings showed partial agreement. The postural tasks showed limited between-group discriminative ability, whereas the MatWalk seems to provide informative gait measures, more than the conventional 10MWT, including subtler impairments in the Mild_PD group. However, the ability of these tasks to distinguish between different levels of disease severity was limited.

For what concerns postural analyses, Ground posture was the only condition showing a statistically significant difference between HS and PwPD for the RMS, a parameter which reflects the magnitude of postural sway. At first glance, this finding may appear counterintuitive, as one might expect more challenging conditions to better discriminate between groups, while in this case the simplest task seems to provide greater discriminatory power. A possible explanation is that the more complex surfaces introduced difficulties not only for PwPD, but also for age-matched healthy participants. As postural task difficulty increases, healthy older adults may experience greater instability and variability, thereby reducing their performance and the differences between groups. The Ground condition may have been therefore sufficiently demanding to reveal disease-related impairments while still allowing for healthy participants to maintain relatively stable performance. This interpretation is supported by the within-group analysis, where the Soft and Root-like surfaces produced significant changes across several postural parameters compared with the Ground condition in HS. These findings indicate that environmental manipulations successfully increased task difficulty even in healthy people. Therefore, the lack of enhanced discrimination between HS and PwPD in the more challenging conditions may not necessarily indicate an absence of disease-related deficits, but rather a reduction in between-group contrast caused by the increased demands imposed on both groups. On the other hand, PwPD had similar performances across conditions, with only the comparisons between Ground and Soft mat and between Ground and Pebbles mat which yielded to statistically significant differences for the Ellipse Area parameter. Possible explanations include greater intra-group variability and that PwPD may already experience considerable postural instability during the Ground condition, thereby limiting the extent to which further environmental challenges produce measurable additional impairments. This phenomenon could be interpreted as a ceiling effect, where baseline deficits constrain the observable impact of increasing task difficulty. Such an interpretation is consistent with previous evidence showing that PD affects the ability to flexibly adapt postural control strategies in response to changing environmental demands [24]. A similar trend was observed where comparing the Mild_PD and the Moderate_PD subgroups, in which no statistically significant differences emerged, likely due to the limited sample size. However, two statistically significant differences (Figure 3b) were observed when comparing HS and Moderate_PD participants: the RMS parameter of the Ground condition was significantly higher in the Moderate_PD group, as well as the Ellipse Area in the Pebbles condition. These findings may reflect greater postural instability and reduced static balance control in participants with more advanced motor impairment, conditions that can substantially increase fall risk and negatively affect quality of life [25]. Altered plantar somatosensory function may also have contributed. Prätorius et al. (2003) reported higher plantar tactile and vibration perception thresholds in PwPD than in HS, with vibration perception thresholds increasing with greater motor impairment, as assessed by the MDS-UPDRS [26]. This association suggests that plantar somatosensory impairment may become more pronounced with increasing disease severity, potentially making the altered sensory input provided by the Pebbles surface more difficult to integrate and contributing to greater postural sway. While this finding alone may not support the use of the current postural protocol as a robust severity discriminator, visual inspection of the graphs further highlights the different abilities of the groups to adapt to the various surfaces, particularly in the RMS parameter, where the different distributions suggest that postural control characteristics vary systematically with disease severity, even when statistical significance is not reached. Interestingly, the Root-like surface appeared to produce similar results across all groups, possibly indicating that this condition imposed comparable challenges regardless of neurological status or that the adopted postural strategies converged under highly demanding conditions. Based on these findings, postural tests performed appear to be insufficiently sensitive to detect the full extent of surface-related difficulties in PwPD. Nevertheless, the fact that HS showed significant differences among conditions suggests that these tasks successfully manipulated postural demands, highlighting their potential usefulness for identifying early alterations in balance control, evaluating functional reserve, or assessing an individual’s capacity to adapt to environmental challenges. 

Walking assessments, on the other hand, provided stronger evidence supporting the value of ecologically relevant challenging tasks. Figure 4a reveals several statistically significant differences between HS and PwPD, particularly during the MatWalk condition. While significant differences in Gait Velocity and Step Length were observed during the 10MWT, the MatWalk task showed relevant findings in all measured gait parameters, suggesting that walking on a soft surface imposes greater challenges than walking on a rigid surface, amplifying gait impairments associated with PD. Walking on a soft surface, in fact, requires continuous adaptation of foot placement, dynamic balance control, and sensorimotor integration [9,27,28]. Such demands may challenge neural systems that are already compromised in PD, particularly those involved in automatic movement regulation and postural adjustment. Consequently, deficits that may remain partially compensated during conventional walking become more evident when environmental complexity increases. Cadence did not differ significantly between groups during the 10MWT. This observation aligns with the known pathophysiology of Parkinsonian gait, often characterized by reduced step length accompanied by relatively preserved or even increased cadence, as patients compensate for shorter steps by increasing step frequency [29]. However, the presence of a significant difference in Cadence during the MatWalk task is noteworthy, as it suggests that increased environmental complexity may interfere with compensatory gait mechanisms, leading to alterations that are not apparent during straightforward walking. Such findings reinforce the concept that challenging walking conditions may reveal latent deficits and provide a more comprehensive evaluation of locomotor function. Moreover, the statistically significant within-group differences in both the 10MWT and the MatWalk in both HS and PwPD further demonstrate the effectiveness of the compliant surface in increasing task difficulty. Notably, the effect appeared particularly pronounced in the Mild_PD subgroup, as shown in Figure 4b, where significant differences emerged across all analyzed gait parameters. This result suggests that individuals in earlier disease stages retain sufficient functional capacity to perform standard walking tasks with relatively minor impairments but experience measurable difficulties when confronted with more realistic environmental challenges.

In contrast, in the Moderate_PD group, only Cadence differed significantly between the 10MWT and MatWalk conditions. As previously noted, this result may partly reflect the limited sample size and increased variability within this subgroup. However, it may also indicate that individuals with more advanced impairment already exhibit altered gait patterns during conventional walking, leaving less room for additional deterioration under more demanding conditions. The number of statistically significant differences identified in the comparisons between HS and the PD disease severity subgroups was relatively limited; however, results should be interpreted cautiously. The absence of significant differences between Mild_PD and Moderate_PD does not necessarily indicate equivalent motor performance but may instead reflect limited statistical power. Future studies involving larger cohorts and a broader spectrum of disease severity will be necessary to clarify the discriminative capacity of these tasks. On the other hand, significant differences emerged in the comparison between HS and Mild_PD (Step Length during the 10MWT and Left Stance Swing Ratio during the MatWalk), as well as between HS and Moderate_PD (Gait Velocity and Step Length during the 10MWT). Nevertheless, the Left Stance/Swing Ratio emerged as a particularly interesting parameter in the present analysis. A significant difference was observed during the MatWalk task between HS and PwPD, and this finding was maintained in the comparison between HS and the Mild_PD subgroup. Since this latter subgroup was characterized by relatively better motor performance and lower disease severity, these findings suggest that the more demanding MatWalk condition may be more appropriate in detecting alterations in gait harmony during the early stages of PD.

As previously discussed, physiological gait is characterized by a stance-to-swing proportion close to the Golden ratio, a feature referred to as “gait harmony” [22]. Alterations in this temporal proportion have been described in PD, with the stance-to-swing ratio deviating from the golden ratio observed in physiological gait [30]. Such deviations contribute to loss of gait harmony described in PwPD, which has been proposed as a quantitative gait benchmark associated with motor impairment severity [30]. Therefore, the sensitivity of the Left Stance/Swing Ratio observed in the present study may reflect its ability to capture alterations in the temporal organization of gait associated with early disease-related motor impairment. Since temporal coordination is closely linked to the neural mechanisms underlying gait automaticity, disruptions in this parameter may provide insight into deficits that are not fully captured by conventional spatiotemporal measures. The finding that this parameter discriminated between HS and individuals with Mild_PD under the more challenging MatWalk condition further supports its potential as an exploratory digital gait biomarker for early gait impairment.

However, the fact that only the Left Stance/Swing Ratio reached statistical significance should be interpreted with caution. Given the limited sample size and the inherent variability of Parkinsonian gait, the observed lateralized effect may partly reflect statistical variability. Alternatively, the unilateral sensitivity of the Left Stance/Swing Ratio may be related to the asymmetric nature of PD. Since motor symptoms frequently progress asymmetrically, alterations in the temporal organization of gait may be more pronounced on the most affected side, resulting in greater deviations from the physiological stance-to-swing proportion. It should be acknowledged that the sample included in this study was mainly composed of patients with bilateral PD in the Moderate group while the Mild group was composed of seven patients with left-side impairment. Further investigations are therefore necessary to better clarify these results.

For what concerns fall history, the proportion of participants reporting at least one fall in the previous year increased across clinical groups, from 12.5% in healthy subjects to 22.2% in patients with mild PD and 42.9% in those with moderate PD. This descriptive pattern is consistent with the progressive increase in fall risk associated with disease severity and may indicate that even relatively subtle impairments in patients with mild PD can have clinically relevant consequences in daily life. However, these findings should be interpreted cautiously given the small and unequal group sizes and should not be taken as evidence of a direct association between disease severity and fall frequency. Some limitations should also be acknowledged. First, the different footwear conditions (shoes/barefoot) represent a potential confounding factor: previous studies in PwPD have reported differences in spatiotemporal gait parameters between barefoot and habitual footwear conditions, including step length and cadence [31,32]. However, the purpose of the comparison was not to isolate the biomechanical effect of surface characteristics under otherwise identical footwear conditions, but rather to investigate motor adaptations under two functionally distinct and ecologically relevant walking conditions.

Second, the shorter path length of the MatWalk may limit direct comparability with the 10MWT and contribute to differences in spatiotemporal gait parameters. Indeed, walking path length has been shown to affect gait characteristics, particularly when very short walking distances are used [33]. This potential effect was partially mitigated by analyzing only the central portion of each test, thereby excluding acceleration and deceleration phases as well as turning periods. Nevertheless, walking bouts were not strictly standardized in terms of walking distance or speed, which may have introduced additional variability between participants. Observed differences between the 10MWT and MatWalk should be interpreted as reflecting the combined effects of several simultaneously changing experimental conditions, rather than as evidence of an isolated effect of the pliable walking surface. Future studies should consider more rigorous standardization of walking distance and speed, or alternatively stratify analyses according to walking speed and path length, to improve comparability and reduce potential confounding when investigating the effects of different surfaces and environmental conditions. Third, although sex imbalance may have influenced the results, the absence of between-group differences in anthropometric characteristics suggests that this potential effect was likely limited. Also, the asymmetric distribution of laterality between groups, with predominantly left-sided involvement in the Mild group and bilateral involvement in all patients in the Moderate group, should be taken into account. Therefore, the generalizability of our findings to patients with unilateral or asymmetric involvement may be limited.

Finally, medication responsiveness represents an additional potential source of variability. Individual responsiveness to levodopa was not systematically assessed during the experimental session; therefore, acute inter-individual differences in medication response could not be directly accounted for in the statistical analyses. 

## 5. Conclusions

Taken together, the findings of this study support the hypothesis that incorporating ecologically relevant motor tasks into a clinical routine assessment can enhance the detection of functional impairments in PD and the potential identification of subtle deficits in health people, capturing distinct characteristics of motor performance under the tested conditions. While postural assessments demonstrated limited sensitivity to disease severity, the walking tasks, particularly those performed on compliant surface, revealed a broader range of deficits and appeared capable of uncovering impairments not evident during conventional testing. These results emphasize the importance of evaluating motor performance under conditions that more closely resemble real-world environments. Any potential biomarker relevance should be confirmed in larger, independently validated cohorts. Future investigations involving longitudinal designs and additional challenging motor scenarios will be essential to determine whether these approaches can contribute to earlier diagnosis, more accurate disease monitoring, and improved assessment of rehabilitation outcomes in Parkinson’s disease. 

## Figures and Tables

**Table 1:** Characteristics of each mat and details of terrain simulation.

Mat Type	Features	Aspect
Soft	A flat surface with a medium elastic response that mimics the characteristics of smooth, sandy terrain.	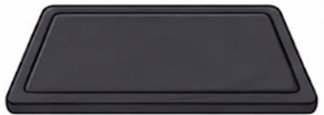
Pebbles	Agglomerates of varying densities, designed to mimic irregular rocky terrain with uniform relief—meaning consistent shapes and sizes throughout.	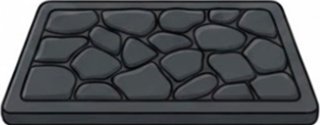
Root-like	Rugged surface simulating uneven, sandy terrain.	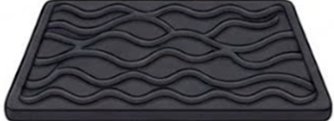

Note: the images displayed on the table were partially AI-modified, to facilitate the description of the surface.

**Table 2:** Mean and standard deviation of demographic and anthropometric characteristics, and clinical scales results for HS, PwPD and the two PD subgroups (*N* = 32).

	HS	PwPD	Mild_PD	Moderate_PD	*p* Value
Age in years (range)	64.63 ± 8 (50–78)	70.00 ± 7 (54–79)	67.89 ± 8 (54–79)	72.71 ± 5 (67–79)	
Body mass, kg	71.51 ± 18 (51.65–108)	75.14 ± 15 (55–105)	75.38 ± 17 (55–105)	74.83 ± 14 (55–90)	
Height, cm	166.20 ± 9 (155–186)	171.10 ± 10 (153.5–186)	173.22 ± 9 (158–186)	167.92 ± 10 (153.5–183)	
Education in years	14.10 ± 4.50	15.13 ± 2.50	14.89 ± 1.90	15.43 ± 3.26	
DGI	22.94 ± 2 **	19.25 ± 4 *	20.33 ± 4	17.86 ± 4 *	<0.001/0.003
Mini-BEST	25.38 ± 3 **	22 ± 4 *	23.67 ± 4	19.86 ± 4 *	0.007/0.013
Disease duration in years (range)	NA	7.67 ± 4.91 (1–18)	7.56 ± 5.36 (1–18)	7.83 ± 4.62 (1–13)	
MDS-UPDRS-Part III	NA	24.31 ± 10	16.67 ± 4 *	34.14 ± 6 *	0.009
Hoehn & Yahr Stage	NA	1.84 ± 0.7	1.56 ± 0.7	2.21 ± 0.4	
1, *n* (%)		5 (31.3%)	5 (55.6%)	—	
2, *n* (%)		8 (50%)	3 (33.3%)	5 (71.4%)	
2.5, *n* (%)		1 (6.8%)	—	1 (14.3%)	
3, *n* (%)		2 (12.5%)	1 (11.1%)	1 (14.3%)	
MDS-UPDRS PIGD-partial axial/gait subscore (range)		2.25 ± 2.25 (0.75–4)	1.33 ± 1.5 (0–4)	3.43 ± 1.4 (2–5)	0.023
LEDD, mg/d (range)		860.43 ± 260.90 (520–1340)	864.29 ± 327.45 (520–1340)	856.57 ± 200.58 (554–1050)	

Note: * = *p* < 0.05, black asterisk = statistically significant difference between HS and PwPD or Mild_PD and Moderate_PD, red asterisk = statistically significant difference between HS and Moderate_PD, LEDD = Levodopa Equivalent Daily Dose.

**Table 3:** Fall characteristics across the three groups.

Fall Characteristic	HS	PD_Mild	PD_Moderate
Past year fall history, *n* (%)			
No falls	14 (87.5%)	7 (77.8%)	4 (57.1%)
History of falls	2 (12.5%)	2 (22.2%)	3 (42.9%)
Total falls in past year, *n* (*M* ± *SD*)	6 (3 ± 2.8)	4 (2 ± 1.4)	3 (1 ± 0)
Injury severity (among fallers), *n* (%)			
Minor (bruises/scrapes)	2 (12.5%)	2 (22.2%)	2 (28.6%)
Severe (requiring medical attention)	—	—	1 (14.3%)
Primary contributing factor, *n* (%)			
Trip hazard	2 (12.5%)	1 (11.1%)	
Balance loss	—	—	1 (14.3%)
While turning	—	—	1 (14.3%)
Room-specific hazard	—	—	1 (14.3%)
More than one cause	—	1 (11.1%)	—
Most common location setting, *n* (%)			
Indoor (house)	—	2 (22.2%)	2 (28.6%)
Outdoor	2 (12.5%)	—	1 (14.3%)

**Table 4:** Missing data across groups and assessment conditions.

Group	10MWT						MatWalk					
	Ellipse Area *	Cadence	Gait Velocity	Step Length	Right Gait Ratio	Left Gait Ratio	Ellipse Area *	Cadence	Gait Velocity	Step Length	Right Gait Ratio	Left Gait Ratio
HS	0	0	1	1	0	0	0	0	0	0	0	0
PwPD	1	1	2	2	1	1	1	2	2	2	2	2
Mild PD	0	0	0	0	0	0	0	1	1	1	1	1
Moderate PD	1	1	2	2	1	1	1	1	1	1	1	1

Note: parameters not reported on the table had no missing values. * = parameter for posturography tasks.

## Data Availability

The data presented in this study are available on request from the corresponding author. The data are not publicly available due to privacy restrictions.

## Abbreviations

The following abbreviations are used in this manuscript:

10MWT	10-Meter Walking Test
DGI	Dynamic Gait Index
H&Y	Hoehn & Yahr
HS	Healthy Subject
IQR	Interquartile Range
LEDD	Levodopa Equivalent Daily Dose
IMU	Inertial Measurement Unit
M	Mean
MatWalk	Walk on a Mat with medium elastic response
Mdn	Median
MDS-UPDRS	Movement Disorder Society–Unified Parkinson’s Disease Rating Scale
Mini-BEST	Mini-Balance Evaluation Systems Test
MMSE	Mini Mental State Examination
Pebbles	Embedded pebbles mat
PIGD	Postural Instability/Gait Dominant
PD	Parkinson’s Disease
PwPD	People with Parkinson’s Disease
RMS	Root Mean Square
Root-like	Root-like surface mat
SD	Standard Deviation
Soft	Medium elastic response mat
